# Convergence in reduced body size, head size, and blood glucose in three island reptiles

**DOI:** 10.1002/ece3.4171

**Published:** 2018-05-20

**Authors:** Amanda M. Sparkman, Amanda D. Clark, Lilly J. Brummett, Kenneth R. Chism, Lucia L. Combrink, Nicole M. Kabey, Tonia S. Schwartz

**Affiliations:** ^1^ Department of Biology Westmont College Santa Barbara California; ^2^ Department of Biological Sciences Auburn University Auburn Alabama

**Keywords:** California Channel Islands, *Coluber constrictor*, *Elgaria multicarinata*, island dwarfism, *Pituophis catenifer*

## Abstract

Many oceanic islands harbor diverse species that differ markedly from their mainland relatives with respect to morphology, behavior, and physiology. A particularly common morphological change exhibited by a wide range of species on islands worldwide involves either a reduction in body size, termed island dwarfism, or an increase in body size, termed island gigantism. While numerous instances of dwarfism and gigantism have been well documented, documentation of other morphological changes on islands remains limited. Furthermore, we lack a basic understanding of the physiological mechanisms that underlie these changes, and whether they are convergent. A major hypothesis for the repeated evolution of dwarfism posits selection for smaller, more efficient body sizes in the context of low resource availability. Under this hypothesis, we would expect the physiological mechanisms known to be downregulated in model organisms exhibiting small body sizes due to dietary restriction or artificial selection would also be downregulated in wild species exhibiting dwarfism on islands. We measured body size, relative head size, and circulating blood glucose in three species of reptiles—two snakes and one lizard—in the California Channel Islands relative to mainland populations. Collating data from 6 years of study, we found that relative to mainland population the island populations had smaller body size (i.e., island dwarfism), smaller head sizes relative to body size, and lower levels of blood glucose, although with some variation by sex and year. These findings suggest that the island populations of these three species have independently evolved convergent physiological changes (lower glucose set point) corresponding to convergent changes in morphology that are consistent with a scenario of reduced resource availability and/or changes in prey size on the islands. This provides a powerful system to further investigate ecological, physiological, and genetic variables to elucidate the mechanisms underlying convergent changes in life history on islands.

## 1 INTRODUCTION

The repeated evolution of multiple traits in independent lineages that reside in the same habitat provides opportunities to identify environmental selective forces as well as constraints on correlated traits across hierarchical levels of organization. Islands are a major habitat type in which to explore such opportunities. Upon the colonization of oceanic islands, newly established island populations may experience reproductive isolation from the mainland, allowing genetic differentiation from ancestral source populations through selection and drift (Grant, 2001). Changes in environmental variables such as temperature, precipitation, and soil nutrients, along with changes in community structure, such as predator, competitor, prey, and parasite diversity and abundance may select for changes in morphology, behavior, and physiology (Bańbura, Blondel, de Wilde‐Lambrechts, Galan, & Maistre, 1994; Buckley & Jetz, 2007; Lindström, Foufopoulos, Pärn, & Wikelski, 2004; Loiseau et al., 2017; Olesen & Valido, 2003; Sagonas et al., 2014; Shine, 1987). For instance, numerous studies have shown that island populations of a wide range of plants and animals differ from mainland populations with regard to traits such as body size, reproduction, dispersal ability, woodiness, and aggression (reviewed in Whitakker & Fernández‐Palacios 2007). Study of physiological differences between island and mainland populations has been more limited, and has focused primarily on tests for differences in immune function with respect to island size and/or parasite diversity (Beadell, Atkins, Cashion, Jonker, & Fleischer, 2007; Lobato, Doutrelant, Melo, Reis, & Covas, 2017; Matson, 2006; Matson & Beadell, 2010; Tompkins, Mitchell, & Bryant, 2006), levels of hormone corticosterone as an index of stress (Müller et al., 2007; Rödl, Berger, Michael Romero, & Wikelski, 2007), digestion efficiency (Sagonas, Pafilis, & Valakos, 2015), and thermoregulatory strategy (Sagonas, Valakos, & Pafilis, 2013).

Both dwarfism (also called nanism) and gigantism, where populations become either significantly smaller or larger than their mainland counterparts, have evolved repeatedly on islands throughout the world in a range of plant and animal species (reviewed in Lomolino et al. 2005). An “island rule” has been postulated, suggesting a trend for gigantism in small species and dwarfism in large species (Foster, 1964; Van Valen, 1973). The island rule appears to hold for a wide range of vertebrates (Boback & Guyer, 2003; Clegg & Owens, 2002; Lomolino, 1985; Lomolino, 2005; Lomolino et al., 2013; Faurby & Svenning, 2016; but see Meiri, Dayan, & Simberloff, 2006; Meiri, 2007; Meiri, Cooper, & Purvis, 2008). Research on the evolution of body size on islands has largely focused on ecological selective forces for body size evolution (Case, 1978; Lawlor, 1982; reviewed in Whitakker & Fernández‐Palacios 2007), and the physiological differences associated with alterations in island body size have not been hitherto investigated. Divergence in physiological mechanisms regulating body size between island and mainland can be predicted to have occurred as they have in laboratory model organisms and agricultural species artificially selected for body size (Borg, Brown‐Borg, & Bartke, 1995; Hauck, Hunter, Danilovich, Kopchick, & Bartke, 2001; Smith, Prall, Siegel, & Cline, 2011; Sumners et al., 2014).

It has been hypothesized that the evolution of dwarfism is a consequence of reduced resource availability on islands, as smaller body sizes may more efficiently be able to survive and reproduce in a low‐resource environment (Lomolino, 1985). Based on our understanding of the physiological consequences of resource restriction in model organisms and humans, small body size on islands is predicted to involve alteration of physiological mechanisms regulating growth and metabolism (e.g., Clemmons & Underwood, 1991; Dunn et al., 1997; Fontana, Klein, Holloszy, & Premachandra, 2006; Roth et al., 2002; Smith, Underwood, & Clemmons, 1995). Blood glucose is a major physiological factor involved in whole‐organism metabolism that is regulated by a feedback mechanism designed to keep levels at or near an average set point, which may vary among species and populations (reviewed in Gangloff et al., 2017; Polakof, Mommsen, & Soengas, 2011; Ruiz, Rosenmann, Novoa, & Sabat, 2002). Vertebrates may obtain glucose either through absorption from digested carbohydrates in the small intestine, or via glycogenolysis (breakdown of glycogen) and gluconeogenesis from noncarbohydrate metabolites. Pancreatic hormones work together to maintain glucose homeostasis, with insulin acting to decrease blood glucose concentrations by facilitating cellular glucose uptake, and glucagon acting to increase blood glucose concentrations by stimulating glycogenolysis and gluconeogenesis (reviewed in Jiang & Zhang, 2003). Within individuals, blood glucose levels are highly plastic with respect to time since eating, typically showing an initial peak at some point after consumption, followed by a gradual reduction over time. Fasting or low‐calorie diets can result in lower blood glucose levels (e.g., Fontana, Meyer, Klein, & Holloszy, 2004; Greene, Todorova, McGowan, & Seyfried, 2001; Kemnitz et al., 1994) and smaller body sizes (e.g., Devlin et al., 2010; Ford & Seigel, 1994; Madsen & Shine, 2000; Mattison, Lane, Roth, & Ingram, 2003). Among taxonomic groups, there may be notable differences in glucose homeostasis. For instance, while blood glucose may decline rapidly in a matter of hours in mammals, it may take days or weeks to reduce glucose to fasting levels in reptiles (Moore, 1967; Gist, 1972; Moon, Owens, & MacKenzie, 1999; McCue 2006). In addition, glucose level set points (basal levels) can be genetically determined and responsive to selection. For example, genetic strains of mice that have a dwarf phenotype also have low blood glucose relative to wild type strains (Borg et al., 1995; Hauck et al., 2001); Angus and Romosinuano cow breeds have lower blood glucose concentration relative to Brahman cows (Coleman et al. 2017); Belgian Blue calves (selected for beef production) have lower blood glucose concentrations relative to Holstein Friesian (selected for milk yield) and East Flemish breeds of calves (selected for milk yield and beef) (Bossaert, Leroy, De Campeneere, De Vliegher, & Opsomer, 2009); and White Plymouth Rock chickens selected for low juvenile body weight have lower blood glucose than those selected for high juvenile body weight (Smith et al., 2011; Sumners et al., 2014). Thus, blood glucose concentrations are determined both genetically and environmentally. Based on these data, we hypothesize that dwarf populations on islands will exhibit lower blood glucose due to either a plastic response to persistent low resource availability over their residence on an island and/or a lower blood glucose set point due to selection in the context of low resource availability.

In a surprising manner, the dynamics of blood glucose in nonmodel organisms have been little explored, and the majority of our understanding of glucose regulation is based on humans and laboratory rodents. In the wild, little is known about how glucose varies with ecological factors, although recent studies suggest that it may vary by year and population (Gladalski et al. 2015; Gangloff et al., 2017; Kaliński et al., 2014; Kaliński et al., 2015; Ruiz et al., 2002). Laboratory studies suggest that pancreatic hormones act to regulate blood glucose similarly to mammals in nonmammalian vertebrates, such as reptiles (Miller & Wurster, 1958; Miller, 1960; Sidorkiewicz & Skoczylas, 1974; Putti, Varano, Cavagnuolo, & Laforgia, 1986; Gangloff, Holden, Telemeco, Baumgard, & Bronikowski, 2016). Furthermore, blood glucose has also been shown to be lower in a fasted state (such as hibernation), and higher with increased food intake in a wide range of reptiles (Haggag, Raheem, & Khalil, 1966; Khalil & Yanni, 1959; Kuckling 1981; Miller & Wurster, 1958; Moore, 1967; Moon et al., 1999; Secor & Diamond, 1997; but see Zain‐ul‐Abedin & Katorski, 1967).

This study begins an investigation of the degree of convergence in body size, head morphology and glucose physiology in three reptile species—two snakes and one lizard— residing in the California Channel Islands found off the coast of southern California, USA. We present evidence of smaller body sizes in island populations of the Santa Cruz Island gopher snake (*Pituophis catenifer pumilis*)—in which dwarfism has already been documented (Klauber, 1946)—as well as for the western yellow‐bellied racer (*Coluber constrictor mormon*) and the southern alligator lizard (*Elgaria multicarinata multicarinata*). As for many islands, species richness is much more limited on the Channel Islands than on the California mainland, which has consequences for prey type and abundance (Whitakker & Fernández‐Palacios 2007; Schoenherr, Feldmeth, & Emerson, 2003). Behavioral observations and analysis of stomach contents of mainland gopher snakes and yellow‐bellied racer snakes indicate that they consume a diversity of prey that vary widely in size, including ground squirrels, pocket gopher, rabbits, mice, voles, woodrats, and numerous snake, lizard, and amphibian species, as well as eggs, nestling birds, and insects (Cunningham, 1959; Klimstra, 1959; Shewchuk & Austin 2001; Rodríguez‐Robles, 2002; Halstead, Mushinsky, & McCoy, 2008). In contrast, for the island populations of both of these snake species, potential prey are smaller and less diverse, being limited to one mouse, three lizards, one frog, and one other snake species, in addition to eggs and nestlings of resident birds (Schoenherr et al., 2003). Reduced prey species richness is also likely for island alligator lizards, which primarily consume invertebrate prey, although average prey size may not differ from the mainland (Cunningham, 1956; Knowlton, 1949). This reduced prey species richness may result in generally reduced resource availability, whether due to increased search times during foraging, or reduced buffering by alternate prey in the case of fluctuations in abundance of primary prey (although note that density compensation within those species that are present may occur—see MacArthur, Diamond, & Karr, 1972; Meiri & Raia, 2010). Furthermore, the preponderance of smaller prey for snakes may also have consequences for head morphology, as relative head size in snakes has been shown to change in association with prey size on islands, depending on the gape‐size required to ingest available prey (Aubret, Shine, & Bonnet, 2004; Forsman, 1991a,b).

In this study, along with presenting evidence for body size divergence, we test for convergent changes in head morphology and metabolic physiology across these three species. If reduced resource availability is indeed a major causal factor underlying the dwarf phenotype, we predict that (a) dwarf snakes will show convergent reductions in head size, as available island prey are smaller than major prey types on the mainland, and (b) all three island reptiles will show lower levels of circulating blood glucose.

## 2 METHODS

### 2.1 Study animals

**Figure 1:**
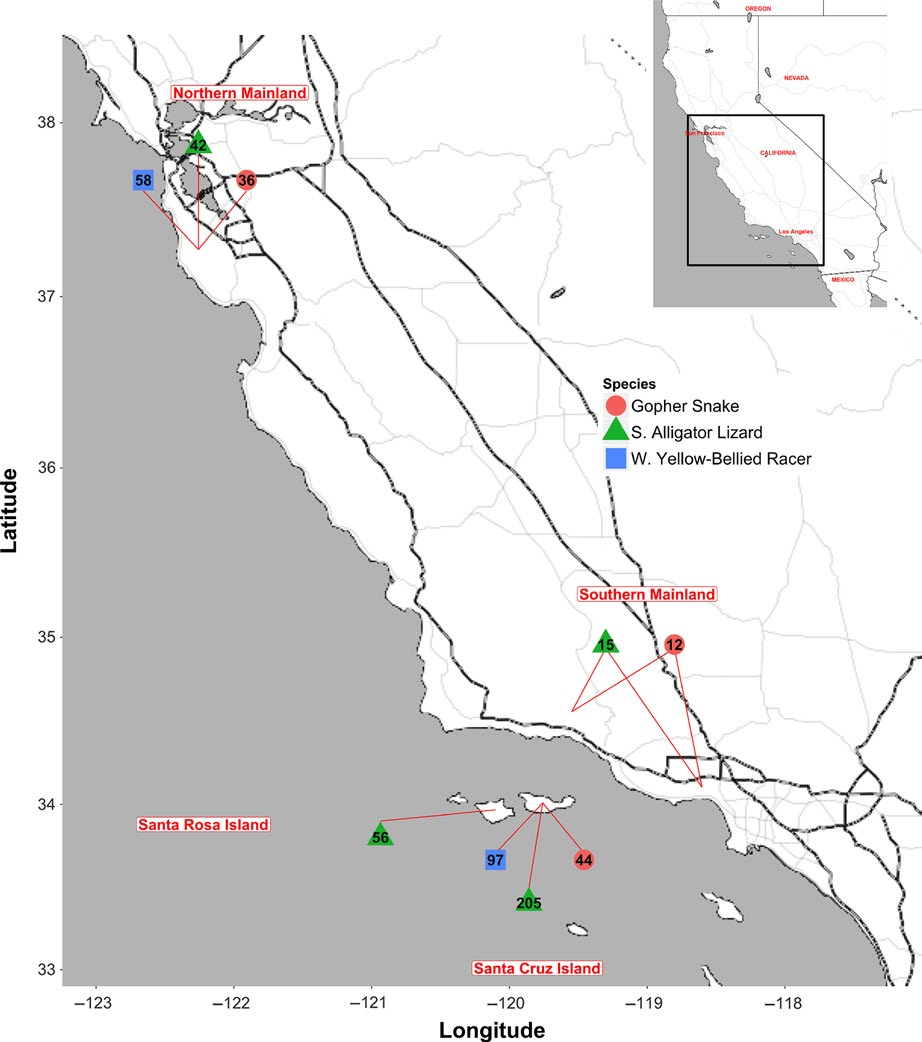
Map of island and mainland collection sites for gopher snakes, racers, and alligator lizards. Sample size indicated within each shape

All three species, gopher snakes (*Pituophis catenifer*), western yellow‐bellied racers (*Coluber constrictor*)*,* and southern alligator lizards (*Elgaria multicarinata*) were captured from both island and mainland populations in California. All procedures involving animals were approved by the Westmont Institutional Review Board and the University of California, Santa Barbara Institutional Animal Care and Use Committee. Island sampling occurred on Nature Conservancy and Channel Islands National Park land on Santa Cruz Island (SCI) (all three species) and Santa Rosa Island (SRI) (alligator lizards only). Mainland sampling occurred at two southern sites that are adjacent to the islands: the Los Padres National Forest (Santa Barbara Ranger District) in Santa Barbara County, and the Santa Monica Mountains National Recreation Area in Ventura and Los Angeles counties (gopher snakes and alligator lizards only); and one northern site, in the Midpeninsula Regional Open Space District in San Mateo County (all three species) that is approximately 475 km north of the southern sites (Figure 1). As western yellow‐bellied racers are rare on the southern California mainland, they were sampled only in the northern site, San Mateo County. Body size and head morphology sampling occurred from 2012 to 2017, and blood glucose sampling occurred from 2015 to 2017 (with the exception of gopher snakes, for whom no data were available for 2017 due to low capture rate in this year). The active foraging/reproductive season when southern California reptiles are most easily captured generally ranges from March to late May. Gopher snakes and alligator lizards were captured in both March (when they emerge from hibernation) and May, whereas racers (which have not yet emerged from hibernation in March) were sampled only in May.

**Figure 2:**
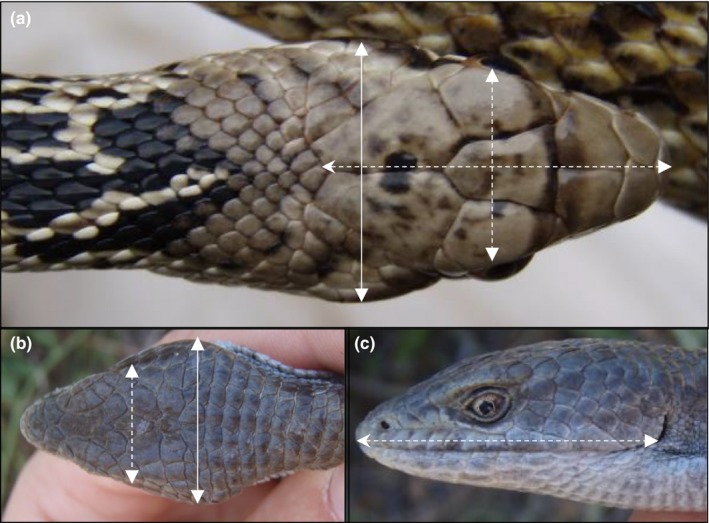
Measurements of head width (dotted vertical lines), width between eyes (solid vertical lines), and head length (dotted horizontal lines) in snakes (a) and lizards (b–c)

All animals were hand‐captured, either while out basking or from under cover objects. Each individual was bled from the caudal vein within 1–10 min of capture, and baseline blood glucose readings of a small drop of blood were taken using a handheld glucometer (FreeStyle Lite by Abbott). Each animal was subsequently measured, sexed, and released at the point of capture. Measurements included snout‐vent length (SVL), head width, width between eyes, and head length (Figure 2). Head width in both snakes and lizards was measured at the widest point of the head. Head length was measured as the distance from snout to ear in lizards, and the distance from snout to the posterior edge of the parietal scales in snakes. Males of both snake species were easily identified by either inspection of the tail morphology and/or eversion of hemipenes. Alligator lizards can be difficult to sex accurately, as young individuals in particular show low dimorphism, and sex probing, which involves using a lubricated probe to determine the presence of hemipenes, is less effective than in other squamates due to the presence of hemiclitores in females (Telemeco, 2015). For 2015–2016, we did not record sex for alligator lizards. However, for 2017, we confirmed female sex in alligator lizards by identifying eggs/follicles via field‐portable ultrasonography (SonoSite M‐Turbo Ultrasound; Fujifilm SonoSite, Inc.), and individuals with markedly triangular heads and less pear‐shaped bodies were classified as males (Beck, 2009; Stebbins, 2003). The sample sizes for each measure are detailed in Supporting Information Table S1.

### 2.2 Statistical analyses

All analyses were conducted using JMP 10.0.0 (SAS Institute Inc.). Effects with a *p‐*value > 0.2 were dropped from the final model, and significant differences among means for main effects or interactions with more than two groups were analyzed using a post hoc comparisons of least square means. For each snake species, body size (SVL) was analyzed using analysis of variance (ANOVA) with the full model containing sampling year, location (Island, Mainland), study site nested within location (Island: Santa Cruz Island (SCI); Mainland: northern, southern sites) and sex as the main effects, as well as a two‐way interaction between location and sex (Gopher snakes: Mainland: *n* = 48; Island: *n* = 44; Racers: Mainland: *n* = 58; Island: *n* = 97; Supporting Information Table S1). For gopher snakes and alligator lizards, two southern sites in the Los Padres National Forest and the Santa Monica Mountains National Recreation Area were pooled due to limited sample sizes, and similarity in trends. For alligator lizards, the same model (although including Santa Rosa Island (SRI) as an additional island site nested within the island location) was applied to only 2017 body size data, for which we had a subset of males and females identified (Mainland: *n* = 19; Island: *n* = 52). However, to expand our sample size despite a lack of information on sex, we also conducted analysis of the full alligator lizard data set (2012–2017; Supporting Information Table S1) with only location as a main effect (Mainland: *n* = 57; Island: *n* = 261). We chose to exclude juveniles (*Elgaria* <60 mm (*n* = 18); *Coluber* <375 mm (*n* = 7); no *Pituophis* juveniles captured) from all of our analyses of body size, as alligator lizards and racers with juvenile coloration (Stebbins, 2003) were found only on the mainland. This may indicate either that we were not sampling the very youngest individuals on the islands, or juvenile coloration has been lost in island populations. However, note that our findings remain the same whether or not juveniles are included in our analyses, suggesting that even if juveniles were oversampled on the mainland, mean mainland body sizes were significantly larger than those of island populations regardless.

Relative head measurements were calculated by dividing each of the three measurements of head morphology—head width, width between eyes, and head length—by SVL. A principal component analysis (PCA) of relative head measurements was conducted to reduce the dimensionality of the data set. ANCOVA analysis of the first principal component (PC1) of relative head measurements was conducted in the same manner as analyses of SVL described above, although in this case SVL was included as a covariate to account for ontogenetic/growth‐related changes in relative head size; interactions between SVL and both sex and location were also considered.

Blood glucose levels between island and mainland populations were analyzed using analysis of covariance (ANCOVA) with location, site nested within location, sex, and year as main effects, SVL as a covariate, and all two‐way interactions. Blood glucose was log10‐transformed to achieve normality. For island gopher snakes and alligator lizards, for which both March and May glucose data were available, preliminary ANCOVAs were conducted to determine whether there was an effect of season on blood glucose. As gopher snakes showed no difference in blood glucose across the season (see Results) data from both months were pooled for the final analysis (Mainland: *n* = 30; Island: *n* = 27). However, as there was an effect of season on blood glucose in alligator lizards (see Results), and we did not have adequate sampling of this species for each year on the mainland in March, we included only May data in our final analysis of blood glucose for this species (Mainland: *n* = 44; Island: *n* = 109). Note, however, that our findings remain consistent whether or not March data is included. As all racers were captured in May, our sample size was not affected by variation in season (Mainland: *n* = 54; Island: *n* = 78).

## 3 RESULTS

### 3.1 Body size

**Figure 3:**
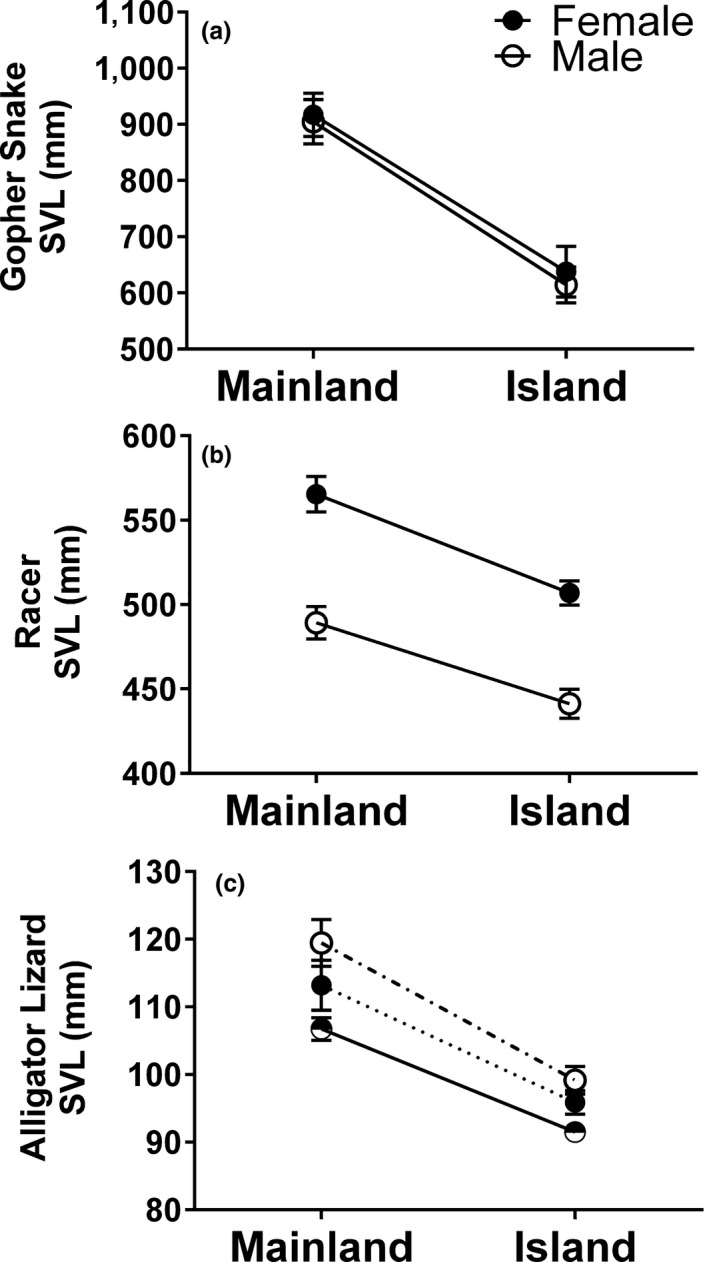
Differences in body size (SVL) between California Channel Island and California mainland populations of gopher snake (a), western yellow‐bellied racer (b), and southern alligator lizard (c). For (c), males and females were pooled (half black/half white symbols) for the full analysis involving all years; however, data for 2017 when sex was known is also shown as dashed lines. Model‐adjusted least square means and standard errors of the means are shown for both males and females, except for alligator lizards, in which case they are pooled

All three species showed significantly smaller body sizes on islands than on the mainland (Figure 3; Table 1). Gopher snakes showed a significant effect of location, with island (SCI) gopher snakes (Males: 614 mm; Females: 637 mm) being 31% smaller on average than mainland gopher snakes (Males: 904 mm; Females: 917 mm) (Figure 3a). Furthermore, a significant effect of site nested within location revealed that while snakes from both mainland sites were significantly larger than SCI; the snakes from the southern sites adjacent to the islands were significantly larger than the northern site snakes in our sample. Gopher snakes had neither significant differences between sampling year, the sexes, nor significant interaction between sex and location. Racers showed both significant effects of location and sex and their interaction, but no effect of sampling year. Within each sex, island (SCI) racers (Males: 441 mm; Females: 507 mm) were smaller than their mainland counterparts (Males: 489 mm; Females: 565 mm), with both males and females being 10% smaller on average (Figure 3b). Alligator lizards from 2017 for whom sex was known showed smaller body sizes on islands (SCI and SRI) for both sexes (Location: *F*_1,64_ = 23.07; *p *<* *0.0001), as well as a nonsignificant trend for males having larger body size than females (Sex: *F*_1,64_ = 3.11; *p *=* *0.083). However, as there was not a significant interaction between location and sex in the 2017 sample (Location × Sex: *F*_1,64_ = 1.01; *p *=* *0.318), we present the analysis of the full data set with all years in Table 1 and Figure 3c, with males and females pooled. A significant difference in body size across sampling years was found, with earlier years (2013–2015) having generally having smaller body sizes than later years (2016–2017). A significant effect of location revealed that alligator lizard was on average 14% smaller on the islands (91 mm) than on the mainland (107 mm).

**Table 1:** Results of ANOVA on of body size (SVL) for gopher snakes, racers, and alligator lizards

Species	Effect	*df*	*F*	*p*	Estimate (*SE*)
Gopher snake	Location	1,91	56.87	<0.0001	−127.21 (45.92)
	Site (Location)	1,91	21.82	<0.0001	–
Racer	Location	1,151	62,04	<0.0001	26.65 (4.55)
	Sex	1,151	34.26	<0.0001	34.87 (4.43)
Alligator lizard	Location	2,316	71.60	<0.0001	−8.78 (0.91)
	Year	4,316	2.69	0.031	–

### 3.2 Head morphology

For all three species, the first principal component (PC1) had high positive loadings for all three relative measures of head morphology (Gopher snake: 0.88–0.89; Racer: 0.85–0.87; Alligator lizard: 0.79–0.81), and explained the majority of the variation in relative head size (Gopher snake: 79%; Racer: 73%; Alligator lizard: 64%). This suggests that individuals with higher PC1 values have larger head sizes relative to their body size.

**Figure 4:**
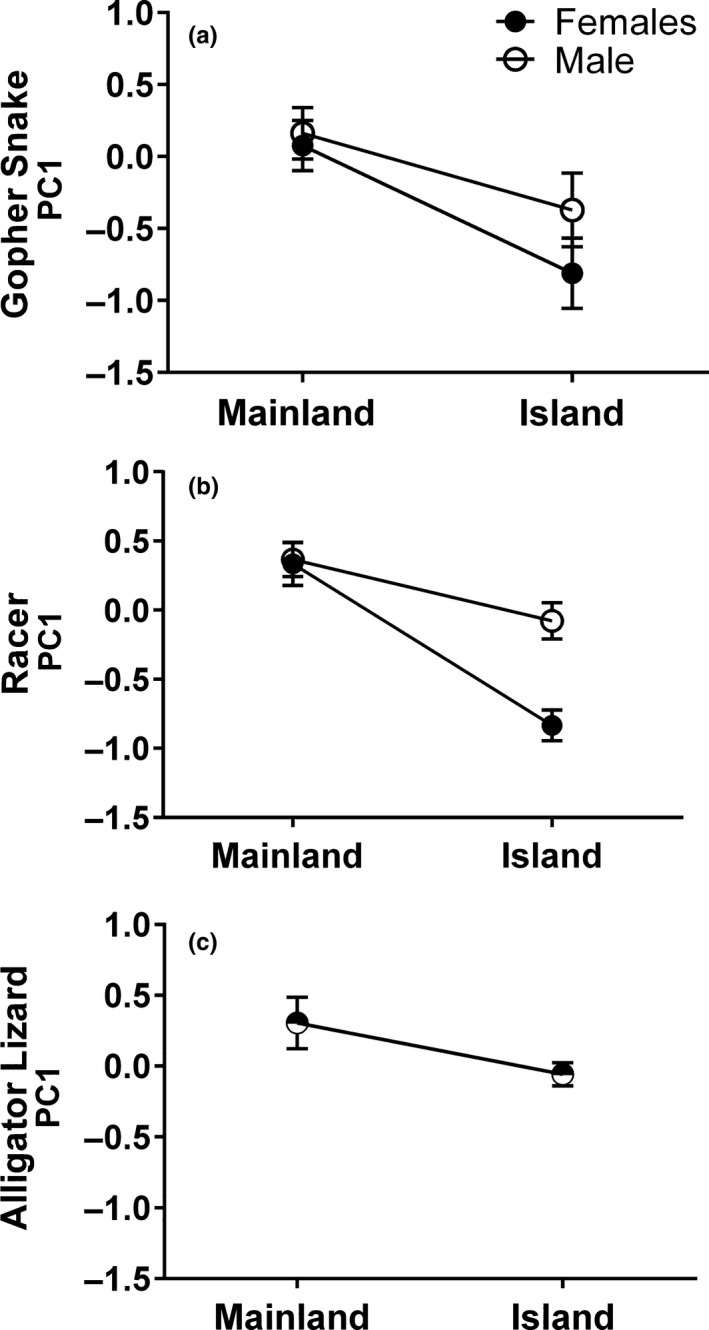
Differences in relative head size between California Channel Island and California mainland populations of gopher snake (a), western yellow‐bellied racer (b), and southern alligator lizard (c). Model‐adjusted least square means and standard errors of the means are shown for both males and females, except for alligator lizards, in which case they are pooled

**Figure 5:**
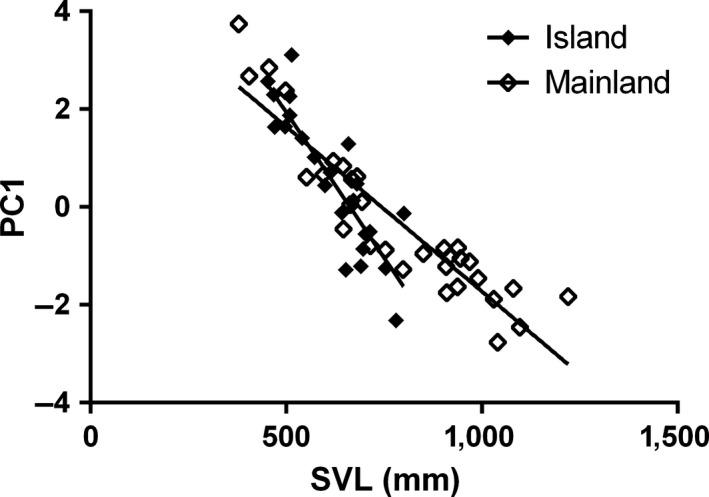
Interaction between the first principal component of relative head size (PC1) and body size (SVL) between Santa Cruz Island gopher snakes and California mainland gopher snakes

All three island reptiles showed evidence of lower PC1 relative to the mainland, although this difference was statistically significant in the two snake species, but only marginally significant (*p *=* *0.075) in alligator lizards (Figure 4; Table 2). Furthermore, all three species had a significant decrease in PC1 with SVL indicating a lower rate of growth of the head relative to the body through ontogeny. As all three head measurements had high positive loadings for PC1, this suggests that relative head morphology is smaller in island reptiles, with smaller head sizes being particularly pronounced in snakes. Gopher snakes also showed a significant interaction between location and SVL, with the slope for island snakes showing a more steep decrease in PC1 with increasing size than mainland snakes (Island slope: −0.012 ± 0.0002; Mainland Slope: −0.0068 ± 0.0005), indicating differences in relative head size during ontogeny in the two locations (Figure 5). Racers showed a significant negative association in PC1 with SVL in both locations, as well as a significant interaction between sex and location. Post hoc comparison of least square means revealed that island females have a significantly lower PC1 then all other groups. This pattern suggests that along with decreased head size on the island, there was an increase in sexual dimorphism in head size on the islands relative to the mainland (Figure 4b). Like both snake species, alligator lizards also showed a significant decrease in PC1 with SVL.

**Table 2:** Results from ANCOVA from the first principal component (PC1) from a principal components analysis of head width, width between eyes, and head length in gopher snakes, racers, and alligator lizards

Species	Effect	*df*	*F*	*p*	Estimate (*SE*)
Gopher Snake	Location	1,50	12.23	0.0010	−0.41 (0.12)
	SVL	1,50	158.87	<0.0001	−0.009 (0.001)
	Location × SVL	1,50	12.12	0.0010	0.003 (0.001
Racer	Location	1,112	37.28	<0.0001	−0.42 (0.07)
	Sex	1,112	7.18	0.0085	−0.20 (0.08)
	Location × Sex	1,112	8.77	0.0037	−0.20 (0.07)
	SVL	1,112	179.29	<0.0001	−0.01 (0.001)
Alligator Lizard	Location	1,326	3.19	0.075	−0.18 (0.10)
	SVL	1,326	27.02	<0.0001	−0.02 (0.01)

### 3.3 Blood glucose

**Figure 6:**
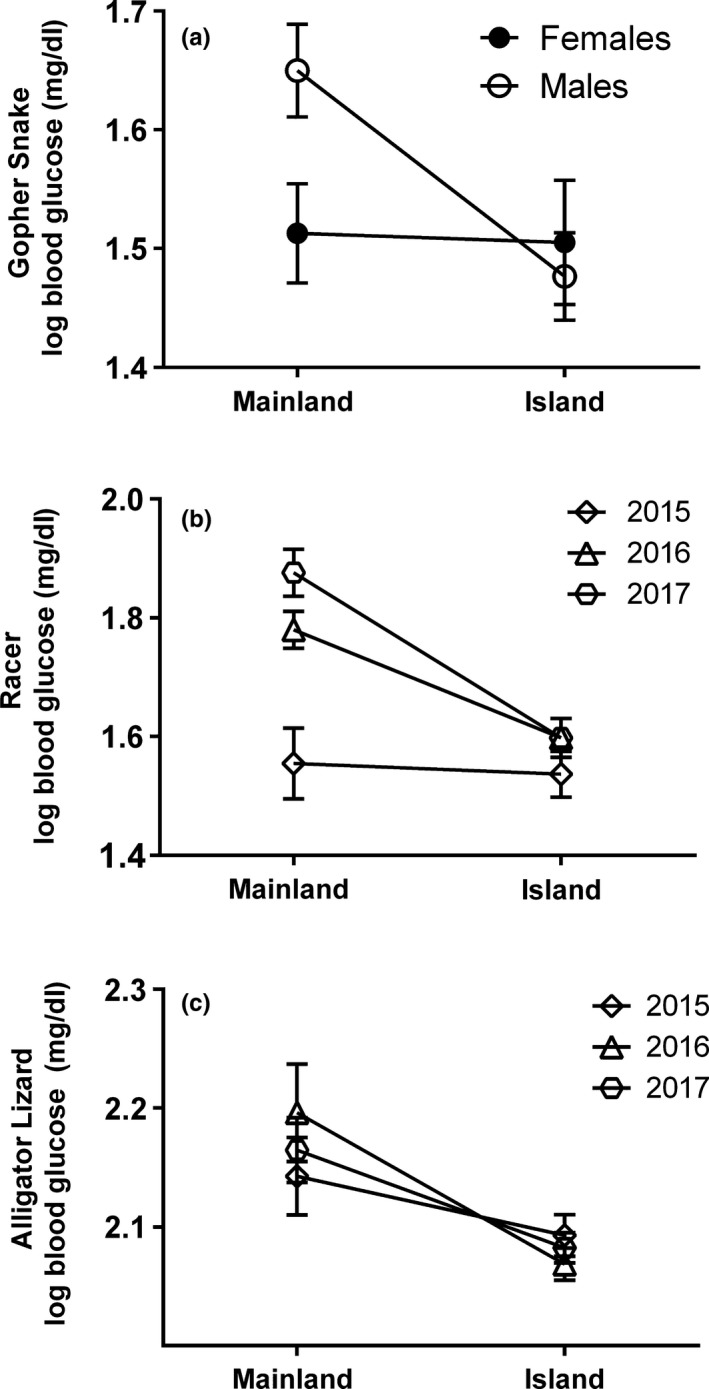
Differences in log10‐transformed baseline blood glucose levels between Santa Cruz Island (SCI), Santa Rosa Island (SRI), and California mainland populations of gopher snake by sex (a), western yellow‐bellied racer by year (b), and southern alligator lizard by year (c). Model‐adjusted least square means and standard errors of the means are shown

All three species showed evidence of lower blood glucose on the islands, although this pattern varied by sex and year (Table 3; Figure 6). An analysis of the effects of season within island (SCI population) sampling revealed that there was no significant difference between March and May blood glucose for gopher snakes (*F*_1,25_ = 1.77; *p *=* *0.195); thus, we included data from both seasons in our full analysis. Blood glucose patterns in gopher snakes did not vary among years (only data for years 2015 and 2016) or with body size. The final model for gopher snake blood glucose contained only location, sex, and their interaction, with a reduction in blood glucose evident in males, but not females (Figure 6a) such that mainland females also had low blood glucose similar to both sexes on the island. Untransformed mean blood glucose in males was 51 ± 4 mg/dl on the mainland in contrast to 31 ± 4 mg/dl on the island, representing a 39% decrease on average.

**Table 3:** Results of ANCOVA on log10‐transformed baseline blood glucose for gopher snakes, racers, and alligator lizards

Species	Effect	*df*	*F*	*p*	Estimate (*SE*)
Gopher snake	Location	1,53	4.44	0.0398	0.04 (0.02)
	Sex	1,53	1.60	0.2114	−0.03 (0.02)
	Location × Sex	1,53	3.73	0.0588	−0.04*0.02)
Racer	Location	1,125	21.015	<0.0001	−0.08 (0.02)
	Year	2,125	9.18	0.0002	–
	Sex	1,125	4.22	0.0419	−0.02 (0.02)
	SVL	1,125	0.16	0.6902	0.0001 (0.0003)
	Location × Year	2,125	4.29	0.0156	–
	Sex × SVL	1,125	12.60	0.0005	0.0009 (0.0002)
Alligator lizard	Location	1,184	32.85	<0.0001	−0.06 (0.01)
	SVL	1,184	2.75	0.0991	−0.001 (0.001)

The final model for racer blood glucose was more complex, showing significant interactions between location and year, and sex and SVL. Island racers had lower blood glucose than mainland racers in both 2016 and 2017, but not 2015. In 2015, glucose levels were roughly equivalent between locations, with mainland glucose matching low island levels from all 3 years (Figure 6b). Untransformed mean blood glucose in 2016 and 2017 was 74 ± 5 mg/dl on the mainland on average in contrast to 44 ± 4 mg/dl on the island, representing a 41% decrease. While blood glucose was on average higher in males than females, the significant interaction with SVL revealed that glucose declines with increasing SVL in females but does not change in males.

An analysis of the effects of season within island (SCI and SRI population) alligator lizard sampling revealed that March did have significantly higher blood glucose than May (*F*_1,200_ = 6.84; *p *=* *0.0096); thus we excluded data from March in our full analysis as we did not have March data from mainland sites. The final model for alligator lizard blood glucose contained only location and SVL, with both island populations of alligator lizards having significantly lower blood glucose than mainland lizards (Figure 6c). Untransformed mean blood glucose was 154 ± 5 mg/dl on the mainland in contrast to the islands, 120 ± 3 mg/dl on SCI, and 120 ± 7 mg/dl on SRI, representing a 22% decrease. A nonsignificant negative relationship between blood glucose and body size was observed. Although there was not a significant interaction between location and SVL, analysis by location indicates that there is a significant decrease in blood glucose with SVL in island (*F*_1,84_ = 4.36; *p *=* *0.040), but not mainland alligator lizards (*F*_1,42_ = 0.011; *p *=* *0.918).

## 4 DISCUSSION

Across the three species, island body sizes ranged from 10% to 31% lower than mainland body sizes. Thus, all three reptiles in our study showed strong evidence of island dwarfism—although, interestingly, island racers that are strongly sexually size dimorphic show parallel reduction in body size within each sex, while for island gopher snakes and alligator lizards both males and females were smaller than their mainland counterparts of either sex (Figure 3). Dwarfism in these island reptiles could be due to plasticity, drift, or selection, or a combination of these. While body size is a highly plastic trait, the geological history of the California Channel Islands suggests that the timeframe of the island population isolation better supports a scenario of evolutionary change. The California Channel Islands were formed as a result of tectonic activity along the western north American coast, and thereby were never connected by land to the mainland after they emerged from the ocean approximately 5 million years ago (Atwater, 1998). The northern Channel Islands are composed of a chain of four islands—Anacapa, Santa Cruz, Santa Rosa, and San Miguel—which until approximately 9,000 years ago together constituted a single continuous island called Santarosae. Fossil evidence from San Miguel indicates that alligator lizards arrived 34,000 years ago or earlier, suggesting that the smaller body sizes we report on both Santa Cruz Island and Santa Rosa Island may represent a single evolutionary event that occurred before these islands became dissociated. The first fossil evidence of gopher snakes occurs in approximately 8,000‐year‐old deposits (Allen, 2013; Guthrie, 1993), while timing of racer colonization of the island is still unknown.

Another Channel Islands dwarf, the Channel Island fox is estimated to have arrived on the islands by rafting or human introduction 9000 years ago, but morphological divergence from the mainland is estimated to have occurred within 2,000 years of arrival (Hofman et al., 2015). The gigantic island scrub‐jay fossils date back to 1 million years on the islands (McCormack, Heled, Delaney, Peterson, & Knowles, 2011), but there is evidence of more recent adaptive divergence in beak morphology within island populations associated with the postpleistocene shift from coniferous to oak forest (Langin et al., 2015). Similar to that morphological evolution on other island systems has been shown to occur relatively quickly after colonization (Aubret, 2015; Vartanyan, Garutt, & Sher, 1993). This study of morphological evolution on both the Channel Islands and other islands worldwide indicate that the timeframes estimated for reptile colonization in our system would allow for ample opportunity for evolutionary divergence in body size from mainland populations.

In addition to smaller body sizes, island populations of all three species showed evidence of smaller relative head sizes—that is, island reptiles had smaller heads for a given size than mainland reptiles. This is consistent with our prediction that head size would be smaller in both snake species, due to limited availability of larger prey on islands (Knowlton, 1949; Cunningham, 1956, 1959; Klimstra, 1959; Shewchuk and Austin (2001). Rodríguez‐Robles, 2002; Schoenherr et al., 2003; Halstead et al., 2008); however, we were surprised to see this trend in alligator lizards as well—albeit marginally significant and less pronounced than that in snakes. Increases in relative head size with larger prey has been documented in both adders (*Vipera berus*) and tiger snakes (*Notechis scutatus*), with evidence that head size can be both a plastic response, and may undergo genetic assimilation over time (Aubret & Shine, 2009; Aubret et al., 2004; Forsman, 1991a,b). The relationship between relative head size and prey size in lizards is less clear. However, there is a general association between larger head size and increased bite force in lizards, which may help reduce handling time of larger or particularly hard invertebrate prey or confer an advantage in aggressive social interactions (Anderson, McBrayer, & Herrel, 2008; Verwaijen, Van Damme, & Herrel, 2002). Thus, it is possible that island alligator lizards have smaller or more easily consumed prey, and/or exhibit reduced aggression, as has been documented in some island fauna living at higher densities than on the mainland (Adler & Levins, 1994; but see Donihue, Brock, Foufopoulos, & Herrel, 2016; Itescu, Schwarz, Meiri, & Pafilis, 2017). Future work in all three species should give more detailed attention to head size and shape, and include characterization of diet, and—in the case of alligator lizards in particular—bite force and social interactions.

In an interesting manner, we found that while both island and mainland populations of all three species showed a negative association in relative head size with body size (i.e., an ontogenetic shift), island gopher snakes had smaller heads at larger body sizes than mainland gopher snakes. This suggests that as island individuals grow, their head size grows at a more reduced rate relative to body size when compared to mainland individuals. Thus, it appears that over ontogeny, island gopher snakes may retain more neotenic head size than mainland snakes of the same size. This is consistent with limited availability of large prey on the islands, as prey size often changes during ontogeny in snakes, with juveniles specializing on small prey, and adults specializing on larger prey (e.g., Lind & Welsh, 1994; Mushinsky, Hebrard, & Vodopich, 1982; Shine, Harlow, & Keogh, 1998).

Furthermore, while relative head size was smaller in island racers for both sexes, there was evidence of increased sexual dimorphism in head size between island males and females. As sexual dimorphism can be a consequence of different foraging strategies, it will be of interest to determine whether diet differs between the sexes for island racers in a manner that would explain differences in head size (Shine, 1991; Forsman, 1991a; Madsen & Shine 1993).

At the physiological level, we found a remarkable consistency in low blood glucose on islands across years for all three species (Figure 6). The mainland alligator lizards had consistently higher blood glucose relative to the islands across all years, but mainland populations of snakes had higher glucose levels that varied across years and populations. Mainland racer populations did show differences among years, as mainland levels were significantly higher than island levels during both 2016 and 2017, but matched island levels during 2015. It is worth noting that even in the two cases where differences between island and mainland glucose were not manifest, mainland levels were down at the consistently low island levels. For gopher snakes, female mainland levels were as low as island levels for both sexes during both years. For racers in 2015, mainland levels were as low as island levels for all 3 years. California experienced a severe drought during our study period, spanning 2012–2016 (Wang, Yoon, Gillies, & Hsu, 2017), with recovery due to high rainfall in more northern locales, including our northern study site, beginning in 2016, and commencing in southern California in 2017. The manifestation of higher levels of blood glucose in racers on the mainland relative to those on the island during 2016 and 2017 may have been due to rapid recovery from drought during these years, associated with greater water and/or prey availability on the mainland. Similar to that it may be that mainland gopher snake females were more severely affected by the drought than males during 2015 and 2016, perhaps due to higher energetic costs of reproduction (note that we do not have 2017 data for this species), and we will see island/mainland differences similar to what we observed in males in the future. Considering it may take days or weeks to reduce glucose to fasting levels in reptiles (Gist, 1972; McCue 2006; Moore, 1967; Moon et al., 1999), and drought is expected to reduce prey availability, the pattern of “island‐like” low glucose levels in the mainland snakes during the drought years is consistent with idea that habitat with reduced prey availability is reflected by lower glucose levels.

In general, lower blood glucose in island dwarf reptiles is consistent with our hypothesis that low resource availability has been a major factor in the evolution of small body size on the islands. However, there are certainly alternative hypotheses that must be weighed carefully. For instance, blood glucose has been shown to increase with stressors such as capture, transport, confinement, handling and trapping, and simulated attack across vertebrates (Barton, 2000; Britton & Kline, 1939; Delehanty & Boonstra, 2009; Harcourt‐Brown & Harcourt‐Brown, 2012; Jessop, Tucker, Limpus, & Whittier, 2003; Lance, Elsey, Butterstein, & Trosclair Iii, 2004; Remage‐Healey & Romero 2001; Strange, 1980; Vijayan & Moon, 1994). Furthermore, corticosterone can rise in response to environmental stressors such as predation risk (e.g., Cockrem & Silverin, 2002; Clinchy, Zanette, Boonstra, Wingfield, & Smith, 2004; Scheuerlein, Van't Hof, & Gwinner, 2001; Narayan, Cockrem, & Hero, 2013). Thus, it is theoretically possible that blood glucose is maintained at lower levels on the islands relative to the mainland due to a reduction in environmental stressors. More research is needed to explore the interaction between corticosterone, glucose, and predation. However, island species experiencing predatory release tend to exhibit gigantism (e.g., Adler & Levins, 1994; Li et al., 2011; Michaux, De Bellocq, Sarà, & Morand, 2002; Olson & Hearty, 2010; Palkovacs, 2003), whereas all three reptiles in our study exhibit dwarfism. This suggests that both the reduction in body size as well as circulating blood glucose levels are likely a consequence of resource restriction rather than predatory release.

If resource availability on the islands is consistently low, it is possible that reduced blood glucose levels on islands are a consequence of plasticity. However, it is also possible that consistently low resource availability on the island is reducing the blood glucose levels through plasticity. Considering the divergence time estimates of 9000–34,000 years, and the consistently low levels across all 3 years, and all three species on the islands, we believe the most likely explanation is that the island habitat has selected for convergent low glucose set points in each of these dwarf populations, similar to what has been demonstrated in dwarf laboratory mice (Borg et al., 1995; Hauck et al., 2001), artificial selection on chickens for body size (Smith et al., 2011; Sumners et al., 2014) and body type among cattle breeds (Bossaert et al., 2009; Coleman, Chase, Riley, & Williams, 2017).

Future work will determine whether the two other lizards that exist on the northern Channel Islands, the Island fence lizard (*Sceloporus occidentalis becki*) and the side‐blotched lizard (*Uta stansburiana*), exhibit similar relationships between body size and blood glucose levels as on the mainland. Note that dwarfism is only one potential means for organisms to respond to low resource availability, as life‐history theory predicts that when resources are limited, trade‐offs can be manifest in a wide variety of means (reviewed in Stearns, 1989). For instance, instead of investing less in body size, organisms may invest less in components of other life history traits such as reproduction and survival. Thus, as we continue to explore the relationship between body size and physiology in island dwarves, it will be of interest to ask these questions in a full life‐history context for both dwarves and nondwarves, incorporating additional measures of physiological variables involved in growth, metabolism, reproduction, and lifespan.

We demonstrate dwarfism and convergent morphological and physiological differentiation in three independent lineages of reptiles within the same ecological context. This is a powerful system, in which to investigate the selective forces in the island habitat. The patterns of divergence across the lineages support the hypothesis that island dwarfism is the result of reduced and/or altered prey communities. Further work will continue to test this hypothesis. Because we see convergence in multiple traits across the lineages, this will also be a powerful system to investigate constraints across hierarchical levels of organization from the genetic networks and physiology to the morphology and ecology.

## AUTHOR CONTRIBUTIONS

AMS and TSS conceived and designed the study. All authors participated in the field work and data collection. AMS analyzed the data. AMS, TSS, and ADC prepared the manuscript.

## DATA ACCESSIBILITY

The data have been archived in Dryad (https://doi.org/10.5061/dryad.k6k2g1q).
