# Supplementary material for: Convergence in reduced body size, head size, and blood glucose in three island reptiles
Source: Ecol Evol. 2018 May 20;8(12):6169–82. doi: 10.1002/ece3.4171 (PMC6024148; doi:10.1002/ece3.4171)
Supplement: Supplementary file 1 [file ECE3-8-6169-s001.docx]

| **Species** | **Location** | **Year** | **Sex** | **n body size** | **n head size** | **n glucose** |
| --- | --- | --- | --- | --- | --- | --- |
| Gopher snake | Island | 2012 | Female | . | . | . |
|  | Island | 2012 | Male | 3 | . | . |
|  | Island | 2013 | Female | 1 | . | . |
|  | Island | 2013 | Male | 3 | . | . |
|  | Island | 2014 | Female | 1 | . | . |
|  | Island | 2014 | Male | 2 | . | . |
|  | Island | 2015 | Female | 6 | 1 | 5 |
|  | Island | 2015 | Male | 7 | . | 7 |
|  | Island | 2016 | Female | 8 | 8 | 4 |
|  | Island | 2016 | Male | 11 | 11 | 11 |
|  | Island | 2017 | Female | 1 | . | . |
|  | Island | 2017 | Male | 3 | 3 | . |
|  | Mainland | 2012 | Female | . | . | . |
|  | Mainland | 2012 | Male | . | . | . |
|  | Mainland | 2013 | Female | . | . | . |
|  | Mainland | 2013 | Male | 1 | . | . |
|  | Mainland | 2014 | Female | 1 | . | . |
|  | Mainland | 2014 | Male | 1 | . | . |
|  | Mainland | 2015 | Female | 4 | . | 4 |
|  | Mainland | 2015 | Male | 7 | . | 6 |
|  | Mainland | 2016 | Female | 11 | 9 | 10 |
|  | Mainland | 2016 | Male | 10 | 9 | 10 |
|  | Mainland | 2017 | Female | 8 | 8 | . |
|  | Mainland | 2017 | Male | 5 | 5 | . |
| Racer | Island | 2012 | Female | . | . | . |
|  | Island | 2012 | Male | . | . | . |
|  | Island | 2013 | Female | 3 | . | . |
|  | Island | 2013 | Male | 1 | . | . |
|  | Island | 2014 | Female | 7 | . | . |
|  | Island | 2014 | Male | 2 | . | . |
|  | Island | 2015 | Female | 11 | . | 11 |
|  | Island | 2015 | Male | 8 | . | 8 |
|  | Island | 2016 | Female | 18 | 18 | 17 |
|  | Island | 2016 | Male | 19 | 20 | 17 |
|  | Island | 2017 | Female | 17 | 17 | 17 |
|  | Island | 2017 | Male | 10 | 10 | 9 |
|  | Mainland | 2012 | Female | . | . | . |
|  | Mainland | 2012 | Male | . | . | . |
|  | Mainland | 2013 | Female | . | . | . |
|  | Mainland | 2013 | Male | . | . | . |
|  | Mainland | 2014 | Female | . | . | . |
|  | Mainland | 2014 | Male | . | . | . |
|  | Mainland | 2015 | Female | 4 | . | 4 |
|  | Mainland | 2015 | Male | 4 | . | 3 |
|  | Mainland | 2016 | Female | 13 | 13 | 13 |
|  | Mainland | 2016 | Male | 18 | 19 | 17 |
|  | Mainland | 2017 | Female | 10 | 10 | 9 |
|  | Mainland | 2017 | Male | 9 | 10 | 9 |
| Alligator lizard | Island | 2012 | . | . | . | . |
|  | Island | 2013 | . | 9 | 2 | . |
|  | Island | 2014 | . | 20 | 21 | 19 |
|  | Island | 2015 | . | 56 | 62 | 23 |
|  | Island | 2016 | . | 82 | 84 | 41 |
|  | Island | 2017 | . | 98 | 102 | 54 |
|  | Mainland | 2012 | . | . | . | . |
|  | Mainland | 2013 | . | . | . | . |
|  | Mainland | 2014 | . | 6 | 2 | 6 |
|  | Mainland | 2015 | . | 14 | 14 | 10 |
|  | Mainland | 2016 | . | 12 | 12 | 10 |
|  | Mainland | 2017 | . | 25 | 30 | 24 |
